# What’s in a Friendship? Partner Visibility Supports Cognitive Collaboration between Friends

**DOI:** 10.1371/journal.pone.0143469

**Published:** 2015-11-30

**Authors:** Allison A. Brennan, James T. Enns

**Affiliations:** 1 Department of Psychology, Simon Fraser University, Burnaby, BC, Canada; 2 Department of Psychology, University of British Columbia, Vancouver, BC, Canada; University of Tuebingen Medical School, GERMANY

## Abstract

Not all cognitive collaborations are equally effective. We tested whether friendship and communication influenced collaborative efficiency by randomly assigning participants to complete a cognitive task with a friend or non-friend, while visible to their partner or separated by a partition. Collaborative efficiency was indexed by comparing each pair’s performance to an optimal individual performance model of the same two people. The outcome was a strong interaction between friendship and partner visibility. Friends collaborated more efficiently than non-friends when visible to one another, but a partition that prevented pair members from seeing one another reduced the collaborative efficiency of friends and non-friends to a similar lower level. Secondary measures suggested that verbal communication differences, but not psychophysiological arousal, contributed to these effects. Analysis of covariance indicated that females contributed more than males to overall levels of collaboration, but that the interaction of friendship and visibility was independent of that effect. These findings highlight the critical role of partner visibility in the collaborative success of friends.

## Introduction

Research in cognitive psychology has recently begun to acknowledge what others have noted since ancient times: humans are social animals by nature (Aristotle, 4^th^ century BCE). Social influences on human cognitive processes are now being studied aggressively in research domains that previously focused primarily on the individual, including attention [1], perception [2], memory [3], and language [4]. The focus of the questions is also shifting, with greater emphasis now being placed on the dynamic social interactions that occur between people and less on the factors that influence social cognition in an individual (see [5–6] for reviews).

These lines of inquiry have demonstrated that not all social interactions are equally rewarding. As Gilbert [7] noted, “it’s not marriage that makes you happy, it’s happy marriage that makes you happy.” Such diversity in outcome is also true of cognitive collaboration in field studies; teams with an intermediate density of social connections amongst collaborators made more successful Broadway musicals than those with weaker or stronger connections [8], teams produced higher impact research when collaborators were geographically closer to one another [9] and when more researchers contributed to the project [10]. In the lab, the success of teams on collaborative tests of intelligence has been associated with the average social sensitivity of team members and their equity in conversational turn-taking, rather than with individual intelligence, group cohesion, motivation, or satisfaction [11]. In a recent study we showed that the efficiency with which two people collaborated on a task of visual cognition was correlated with the strength of their pre-existing friendship and their equity in communication [12].

While these studies establish important associations between aspects of social interaction and collaborative success, it is important to note that they do not test the direction of the relationship. That is, the foregoing studies did not test whether the quality of the social interaction influenced the success of collaboration, or whether collaborative success influenced the quality of the social interaction, or even whether a third factor influenced both social interaction and collaborative success.

Here we use an experimental design to test whether the quality of social interaction between friends (versus non-friends) and partner visibility influence collaborative success. Study participants were assigned randomly to collaborate with either a friend or a non-friend (i.e., the friend of another participant). One half of these teams were further assigned randomly to work together while separated by a partition that prevented pair members from seeing one another. Not being see one another meant that these pairs could not communicate using body language, such as eye contact, gesture, and posture. The remaining one half of the teams collaborated while partners were fully visible to one another.

We focused on the possible effects of friendship and partner visibility, using a randomized between-subject design, because previous research provides inconsistent evidence about the directional relations among these factors and collaborative success. With regard to friendship, research suggests that friendship influences group productivity, but some studies report greater group productivity among groups with stronger preexisting friendships [13–14], while others report that stronger friendships are linked to reduced productivity [15–16]. However a meta-analysis suggests the opposite structure, reporting that successful group performance influences cohesiveness more than group cohesiveness influences performance [17]. Complicating the interpretation of these relations even further, it is possible that a third factor such as social intelligence [11] may mediate both good rapport and successful group performance.

The direction of the relation between communication and collaborative success is equally unclear. Previous research reports a trading relation between the channel of communication and collaborative success. Collaborative visual search was faster than individual search when pairs communicated verbally, and also when they communicated nonverbally using a gaze cursor that displayed each person’s eye movements to their partner. However search efficiency was impaired, relative to these conditions, when pairs were given the opportunity to communicate both verbally *and* nonverbally [18–20].

Previous research has also reported mixed results on the role of partner visibility in collaborative success. Participant pairs who were visible to one another while collaborating on a navigation task outperformed those who were not [21]. However when pair members were able to see each other via video-mediated communication during collaborative problem solving, it did not improve performance to the same extent as face-to-face interaction [22]. On the other hand, a comparison of online texting versus face-to-face interactions on a variety of group tasks of intelligence showed no advantage for the face-to-face condition [23].

Aside from the trading relations between the channels of communication described above, research has shown that verbal communication is consistently associated with collaborative success. In some past research, the opportunity to communicate verbally during collaboration was reported to be more important than accuracy feedback [24]. When teams performing a perceptual detection task were allowed to freely discuss their decisions, they were more efficient than when they used a numerical scale to communicate their confidence [25]. Moreover, team members that used a similar task-relevant vocabulary were more efficient than teams who communicated using different descriptors and figures of speech [26].

In addition to experimentally testing the direction of the relation between friendship, the role of partner visibility, and collaborative success, we thought it was also important to monitor the possible role of social facilitation in collaborative efficiency. Social facilitation is the tendency for performance to improve when a task is completed in the presence of others; it has been shown in hundreds of studies (for reviews see [27–28]). Drive theorists (e.g., [29]) have argued that physiological arousal underlies this effect, such that the mere presence of another person heightens an individual's arousal, leading to improved performance. In the present study we therefore monitored the psychophysiological arousal of participants in all conditions. Skin conductance response (SCR) and heart rate (HR) were our indices; SCR measured sympathetic affective arousal and HR measured more generalized arousal and bodily state [30]. We noted that HR is also influenced by factors other than arousal such as verbal speech, which was used by participants during team performance, but not individual performance [31]. These measurements were intended to reveal the role of HR and SCR, if any, in the conclusions drawn on the roles of friendship and partner visibility in the efficiency of cognitive collaboration.

It is important to note that we measured HR and SCR, not because we thought they would mediate the effects, but because we wished to rule them out as intervening variables. For this reason, it was also important to show that these measures moved, as they should, when participant arousal was artificially elevated with a startle manipulation. This manipulation check provided necessary context for the null results we expected would occur for HR and SCR in our four experimental groups. Our guiding hypothesis was that the collaborative advantage results from an efficient division of the cognitive load of the task between team members, not from social facilitation mediated by arousal. We used analysis of covariance to consider the role of arousal in the potential influences of friendship and partner visibility on cognitive collaboration. We also used the same approach to consider the gender composition of teams (coded as 0, 1, or 2 females) and their potential contributions to these interactions.

Research has shown that visual search is effectively limited to one item at a time [32]. Therefore performance should improve when two people divide the search task between them such that each person looks for a different target. Of course team members must also coordinate how they will share the task and integrate their individual efforts prior to their joint response, which are processes that require effort and time. We hypothesize that collaborations will be most successful when partners are able to communicate their unique information quickly and efficiently to one another, while at the same time focusing their attention on the visual search task at hand. Friendship should facilitate collaboration through pre-existing efficient channels of communication; partner visibility should aid further by allowing some of that communication to be nonverbal.

## Methods

### Participants

Thirty-seven University of British Columbia students registered for the study using an online research participation system. During registration participants provided the name and email contact of a friend who was also registered in a psychology course and seeking to earn extra course credit. This created a pool of seventy-four total study participants. To test the influence of friendship on team performance, 40 participants were randomly assigned to participate with the friend they indicated during registration (30 female, 10 male; age mean = 20.50), and 34 participants were randomly assigned to participate with the friend that another participant had indicated during registration (16 female, 18 male; age mean = 20.85). This created 20 pairs of friends and 17 pairs of non-friends using the same recruitment method. Four pairs were male-male, 20 pairs were female-male, and 13 pairs were female-female. We selected this sample size with reference to Brennan and Enns [12], who reported a significant association between friendship strength and collaborative efficiency in 22 pairs of friends. Eleven of the pairs tested had partial data loss (missing behavioral data: N = 1 female-female pair of friends; incomplete video data: N = 4; psychophysiological recording errors: N = 6). Table 1 shows the gender composition of the four conditions in the experiment, after randomly assigning participants to these conditions and omitting teams with missing behavioral data. All participants provided written informed consent and were debriefed in accordance with APA guidelines. The University of British Columbia Behavioural Research Ethics Board (H09-01732) approved this research.

**Table 1:** The composition of teams in the study for the four experimental conditions (columns) and gender (rows).

	Visible/Non-friends	Visible/Friends	Partition/Non-friends	Partition Friends	Total
**Female-Female**	2	6	0	4	12
**Female-Male**	5	4	7	4	20
**Male-Male**	1	0	2	1	4
**Total**	8	10	9	9	36

### Partner visibility

To test whether the critical communication channel in team performance was verbal or nonverbal, one half of pairs of friends and non-friends were randomly assigned to complete the team task with a partition between them that prevented partners from seeing each other. The remaining one half of pairs of friends and non-friends completed the task with full sight of their partners. The partition was a standard office divider (108 cm X 149 cm X 4 cm). Pairs in both partner visibility conditions had equal ability and opportunity to communicate verbally because the partition did not interfere with sound transmission nor alter the procedure of the task. The data showed that team members interacted even when they were not visible to each another because they communicated verbally (see Verbal communication below).

### Search displays, apparatus, and procedure

**Fig 1:**
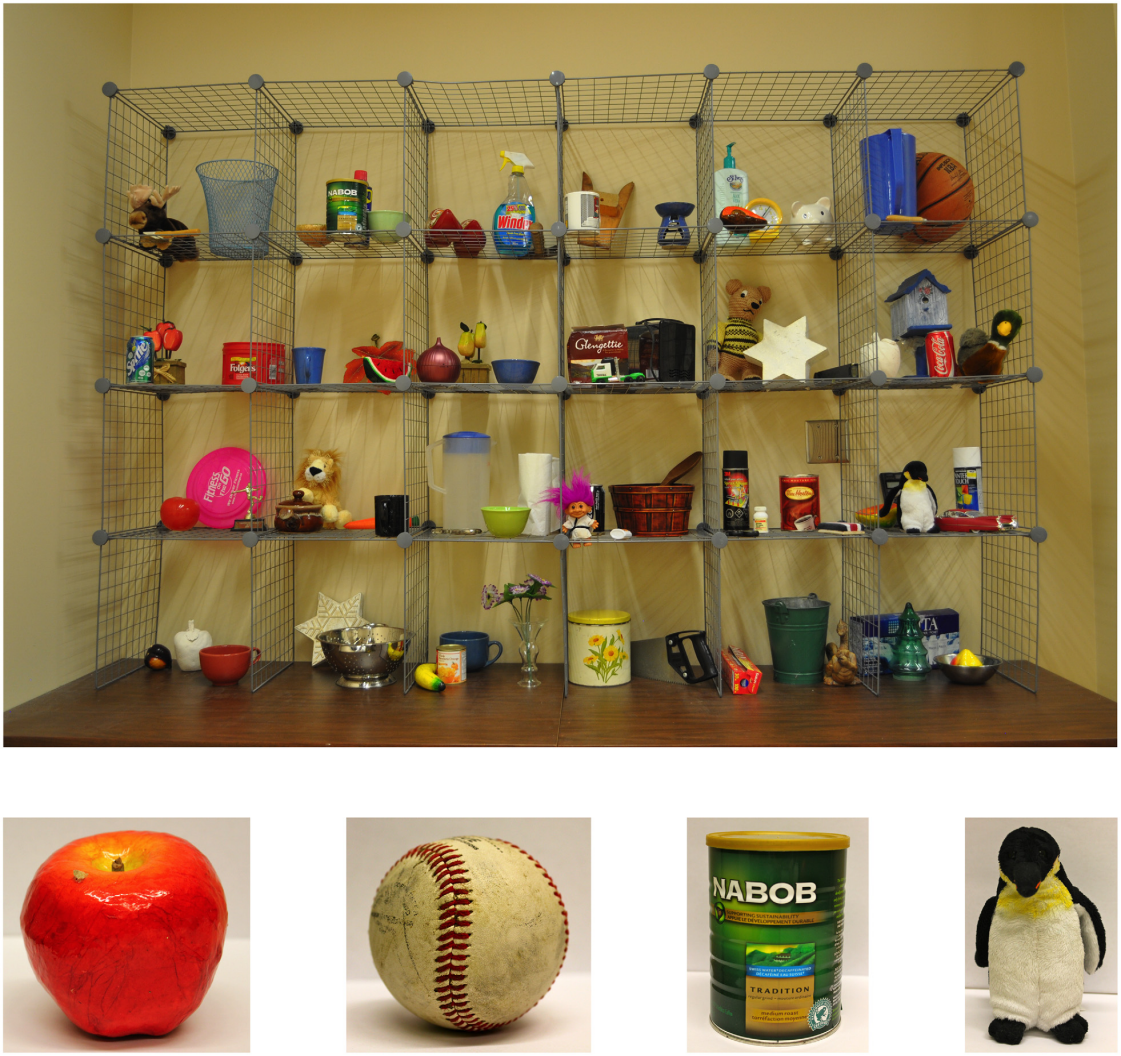
A typical display in the study (top) and the four targets that could appear in each display (bottom). Participants searched displays and indicated whether 0-, 1-, or 2-targets were present. This display contains 2 targets: coffee can and penguin.

As shown in Fig 1, experimental displays depicted wire shelving containing 82 distractor objects commonly found in a home or office and 0, 1, or 2 of 4 possible targets. The same target never appeared twice in a display and each appeared equally often in each quadrant. Distractor objects appeared in four different configurations. This generated 356 displays: 4 without a target, 64 with one target, and 288 with two targets. Sessions were 60 trials in length: 20 trials each with 0, 1, and 2 targets. Search displays for each session were selected using weighted random sampling of the 356 total search displays. Displays subtended 40° x 32° visual angle on a 24-inch iMac computer (screen resolution 1920 X 1200 pixels). The experiment was controlled by Matlab 2010a software and Psychtoolbox3.

**Fig 2:**
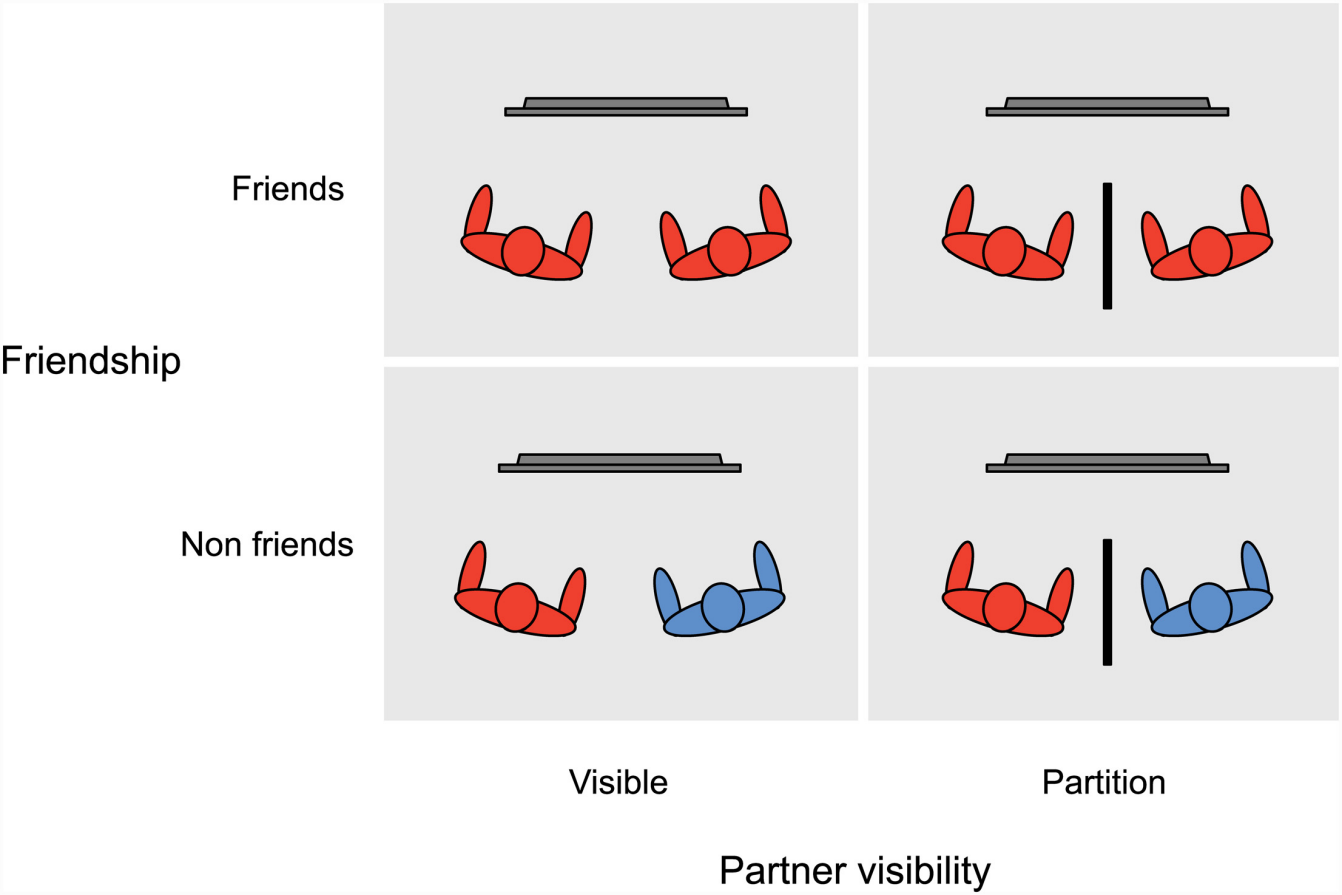
Bird’s eye view of the four experimental conditions (friendship X partner visibility). The arrangement of collaborative participants relative to search displays (gray horizontal bars) and partitions occluding visibility of the partners (black vertical bars).

Participants completed 60 trials alone and 60 trials as a team with another participant who was either a friend or non-friend (see Participants above for additional detail). A partition that prevented nonverbal communication separated one half of the teams of friends and non-friends. Shown in Fig 2, this created four experimental conditions: friends/partition, friends/visible, non-friends/partition, and non-friends/visible. A randomly selected one half of pairs in each of these conditions first completed a session alone before completing a session together as a team, while the other one half first completed a session together as a team before completing a session alone.

During individual and team sessions participants indicated as rapidly and accurately as possible the number of targets present in a display by pressing keys labeled 0, 1, and 2. The four possible targets remained visible throughout the sessions in pictures placed underneath the computer screen. Participants received feedback on their percentage of correct responses every 15 trials. Before beginning the experiment, they were told that sessions were video recorded for the purpose of knowing where they looked while searching. At debriefing we requested that the video recordings be used to analyze their verbal communication; all participants agreed to this request.

The procedure of individual and team sessions differed in only two ways. Teams were instructed to use whatever strategy they thought was best to work together, while individuals were instructed to use whatever strategy they thought was best. Because there was only one keyboard for responses during the team task, team members each took a turn by exchanging the keyboard after 30 trials. During individual sessions, participants each had their own keyboard and computer.

### Verbal communication

To explore whether verbal communication contributed to the effect of friendship or partner visibility on team performance, a research assistant who was naïve to the experimental hypotheses transcribed participants’ verbal communication. Transcription was completed from a video recording (Logitech HD Pro Webcam C920, 1080p) of participants, filmed while they completed the team task. Not all of the verbal communication was linguistic, and utterances such as “Ummm,” “Uh huh,” and “Umph” were included in the transcript and the following measures.

The verbal communication value was computed by counting the number of distinct utterances (i.e., words and non-linguistic utterances such as “uh huh”) made by each team member. For example, the following exchange received a verbal communication value of 12:

**Person A:** “Do you see anything?”

**Person B:** “Yep. Penguin.”

**Person A:** “And apple.”

**Person B:** “Great. So two targets.”

Verbal communication values measured the total number of distinct utterances made by team members from the start of trial 1 to the end of trial 60 during the team task. Teams spoke an average of 664.91 (SE = 46.13) utterances. Higher verbal communication values indicate that team members spoke more to one another.

### Psychophysiological measurement

To explore whether psychophysiological arousal contributed to the influence of friendship or partner visibility on team performance, heart rate (HR) and skin conductance response (SCR) were recorded during both the individual and team tasks. A BVP-Flex/Pro sensor (Model SA 9308M L5890) recorded HR from blood volume pressure (BVP) and two SC-Flex/Pro sensors (Model SA 9309M) recorded SCR. All three sensors (manufactured by Thought Technology Ltd.) recorded from participants’ left hand: HR from the palmar surface of the distal phalanx of the middle finger and SCR from the palmar surface of the distal phalanx of the index and ring fingers. Participants were instructed to minimize hand movements to avoid movement artifacts in the recordings. The sampling rate of the digitized signals of the HR and SCR sensors was 2048 Hz and 256 Hz, respectively.

As described in the introduction, we hypothesized there would not be a difference in HR or SCR between friendship and partner visibility conditions. Therefore we used an auditory startle as a manipulation check, to assess the sensitivity of our psychophysiological measures to an event that should reliably increase arousal [33–34]. An auditory startle (110–115 dB; 1000 Hz; 40 msec) followed trial 39 in both the individual and team tasks. Because both individuals did not complete trial 39 at the same time, the startle sounded after one randomly selected individual completed trial 39. Before beginning the experiment participants were informed that a loud noise would be played at two random times during the experiment.

Data are available as a Supporting Information file (see S1 Dataset).

## Results

We report the results of this study in three parts. First, collaborative efficiency was determined by comparing each pair’s team performance with a model of the optimal performance of the same two individuals working independently, following the analysis procedure used by Brennan and Enns [12, 35]. Second, we investigated whether friendship and partner visibility influenced collaborative efficiency, and whether verbal communication was associated with collaborative efficiency. Third, we tested whether psychophysiological arousal contributed to the effect of friendship and partner visibility on collaborative efficiency.

### Two-person team performance exceeded an optimal performance model of the same two independent individuals

Following Brennan and Enns [12, 35], individual and team performance was compared using Miller’s Race Model Inequality (RMI) [36–37]. This model tested whether the efficiency advantage of teams over individuals was due to a statistical advantage or to collaboration between team members by comparing the distributions of correct response times (RTs) during individual and team performance. The steps of this analysis are described below.

#### Correct RT and accuracy

Before assessing collaborative efficiency using Miller’s RMI, correct RT and accuracy were tested in a mixed-design analysis of variance (ANOVA) with social condition (slower individual, faster individual, team), friendship (friends, non-friends), partner visibility (visible, partition), and task order (first, second) as between-groups factors, and target number (0, 1, 2) as a repeated measures factor. All reported *p*-values have been corrected for violations of sphericity where appropriate, using the Greenhouse-Geisser correction. All interaction effects were tested post-hoc using Tukey’s HSD procedure.

Two people working together were faster and more accurate in the visual enumeration task than either individual working alone. Correct responses were made in an average of 11.99 sec (SE = 0.347). This analysis revealed that team enumeration was faster than enumeration by either the slower or faster individual [F(2, 84) = 41.95, p < .001, η_p_^2^ = .50]. It also showed that enumerating 2 targets was faster than enumerating 1 or 0 targets [F(2, 168) = 177.11, p < .001, partial η_p_^2^ = .68]. The task done second was also significantly faster than the same task done first, [F(1, 84) = 86.36, p < .001, η_p_^2^ = .51].

There was a significant interaction such that the difference between team and individual RT was greater with 0 and 1 targets than with 2 targets [F(4, 168) = 8.19, p < .001, partial η_p_^2^ = .16]. No other effects were significant at p < .05, including the effect of friendship or partner visibility. Most important was the absence of significant interaction involving task order, indicating that the task done second was completed approximately 4 s faster than when it was done first, but that the size of this benefit did not vary with social condition.

Response accuracy was high at 86.12% (SE = 1.07). An ANOVA with the same factors used to examine correct RT revealed that accuracy declined as target number increased [F(2, 168) = 29.45 p < .001, partial η_p_^2^ = .26] and that the task done second was approximately 7% more accurate than the task done first [F(1, 84) = 23.86 p < .001, η_p_^2^ = .22]. Task order did not interact significantly with social condition [F(1, 84) = 1.38, p = .258, η_p_^2^ = .03], indicating that the benefits of teamwork were not dependent on testing order. No other effects were significant at p < .05, including the effect of friendship or partner visibility.

A comparison of the correct RT and accuracy data pointed to a speed accuracy tradeoff involving target number. Participants generally made more rapid responses to 2 targets than to 0- and 1-targets (a mean reduction of 5 seconds in RT) at the expense of accuracy (a reduction in accuracy of 7.5%). Because of this, we submitted the high accuracy RTs in the 0- and 1-target conditions to an RMI analysis of correct RT. This included forty trials of data for each friendship X partner visibility condition, minus the small number of trials in which an error occurred. The results that follow do not differ in any important way when we included the more error-prone data in the 2-target condition.

#### Collaborative efficiency

The algorithm and MATLAB routines provided in [37] were adapted to compare team performance to the optimal performance of the same two individuals alone. This method of calculating collaborative efficiency is ideal because it parses the statistical advantage of teamwork from the collaborative advantage of teamwork [12, 35]. Collaborative efficiency was calculated in 3 steps. First, cumulative density functions (CDFs) of each team’s correct RTs were generated. Each CDF contained a total of forty correct RTs, less the small number of errors that were committed. Second, CDFs of the optimal performance of two individuals alone were generated by combining individual team member’s correct RTs into one distribution, and truncating this distribution at the number of RTs in the collaborative team CDFs. Third, a collaborative efficiency value was generated for each team by subtracting the two-person team performance CDF was from the CDF of the optimal performance by two individuals alone. The difference between these distributions indicates the extent of the performance improvement that resulted from collaboration between team members. Positive values indicate that two-people performed better as a team compared to the model of their optimal performance as two individuals, whereas negative values indicate that the optimal performance model of two individuals surpassed the performance of two-person teams.

Multiple Bonferroni-corrected paired sample t-tests at 10 percentiles along the CDFs indicated that two-person team performance exceeded a model of the optimal performance of the same two individuals at the first seventh percentiles, i.e., .05 through .65 [t(35) = 3.83, 4.32, 4.08, 3.70, 3.48, 2.81, and 2.40, respectively, all ps < .02]. The following analysis of collaborative efficiency uses the values (in msec) of each team at the first percentile tested (i.e., fast RTs at the .05 CDF), since the distinction between team performance and the optimal individual performance model was greatest here. The same pattern of results reported below was also obtained when further percentiles were included; the strength of the effects simply declined.

### Partner visibility supports cognitive collaboration among friends

**Fig 3:**
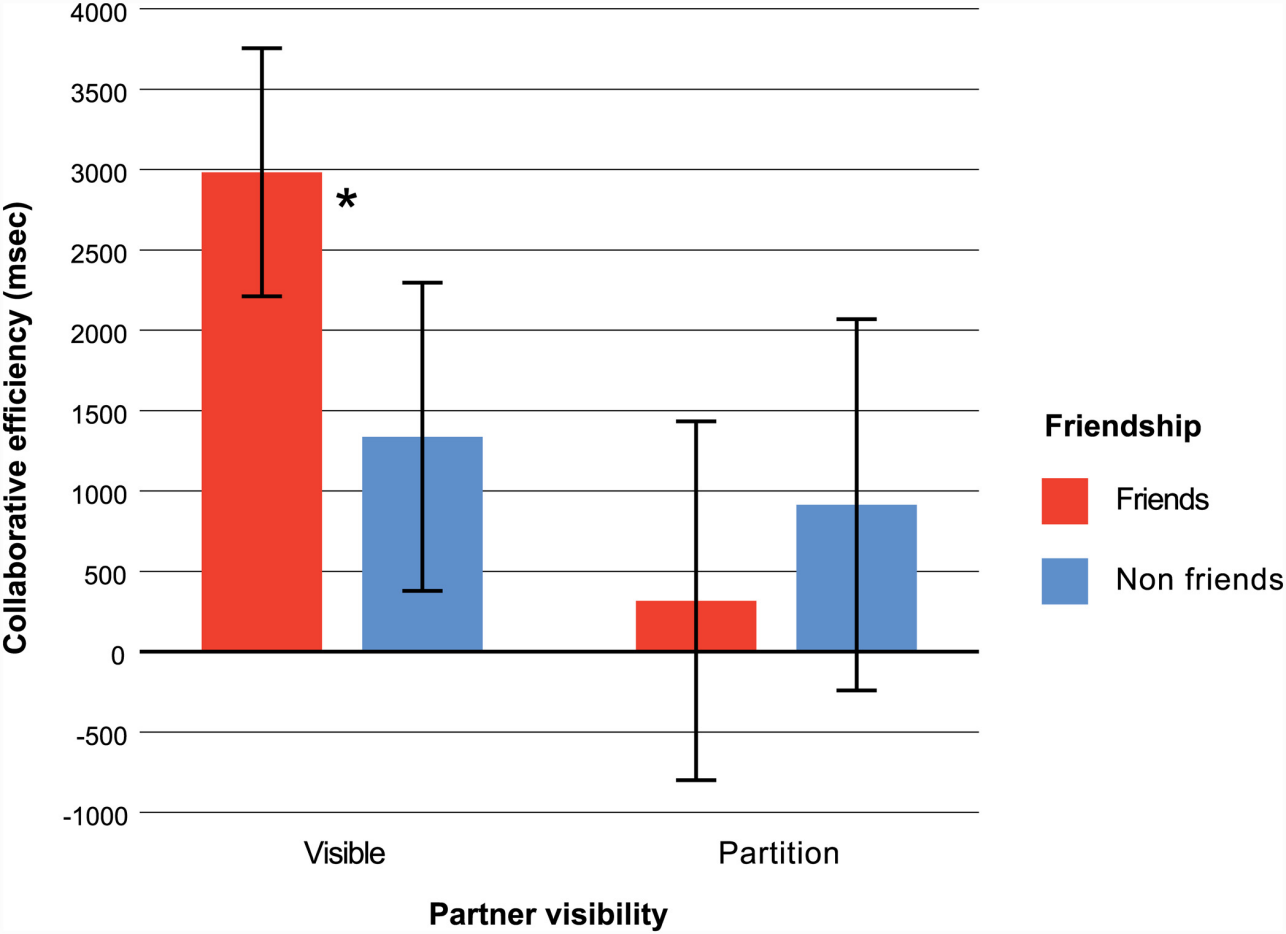
Mean collaborative efficiency as a function of friendship and partner visibility. Collaborative efficiency values index the difference between team performance and the optimal individual performance model (in msec). Friends collaborated more efficiently than non-friends, but only when they were visible to each other. Error bars represent 95% confidence intervals around each mean. The asterisk denotes a significant interaction between friendship and partner visibility.

The effects of friendship (friends, non-friends) and partner visibility (visible, partition) were tested with a 2 X 2 between groups ANOVA. As shown in Fig 3, teams were more efficient when visible to one another than when a partition prevented team members from seeing each other [F(1, 68) = 10.45, p = .002, η_p_^2^ = .13]. Importantly, partner visibility and friendship interacted such that friends collaborated more efficiently than non-friends, but only when they were not separated by a partition [F(1, 68) = 5.50, p = .022, η_p_^2^ = .08]. When partner visibility was prevented with a partition, the efficiency of collaboration between friends and non-friends did not differ.

To test whether the interaction we observed between partner visibility and friendship was influenced by the gender composition of the teams, we repeated the analysis of collaborative efficiency, but this time entering gender as a covariate. We coded each team as containing 0, 1, or 2 females. The covariate of gender was significant on its own, F(1, 67) = 4.37, p = .040, η_p_^2^ = .06, indicating that females contributed more than males to the overall collaborative benefit. But this gender effect was not itself responsible for the interaction we observed between friendship and partner visibility, which remained significant after the covariate had been taken into account [F(1, 67) = 6.67, p = .012, η_p_^2^ = .09].

**Fig 4:**
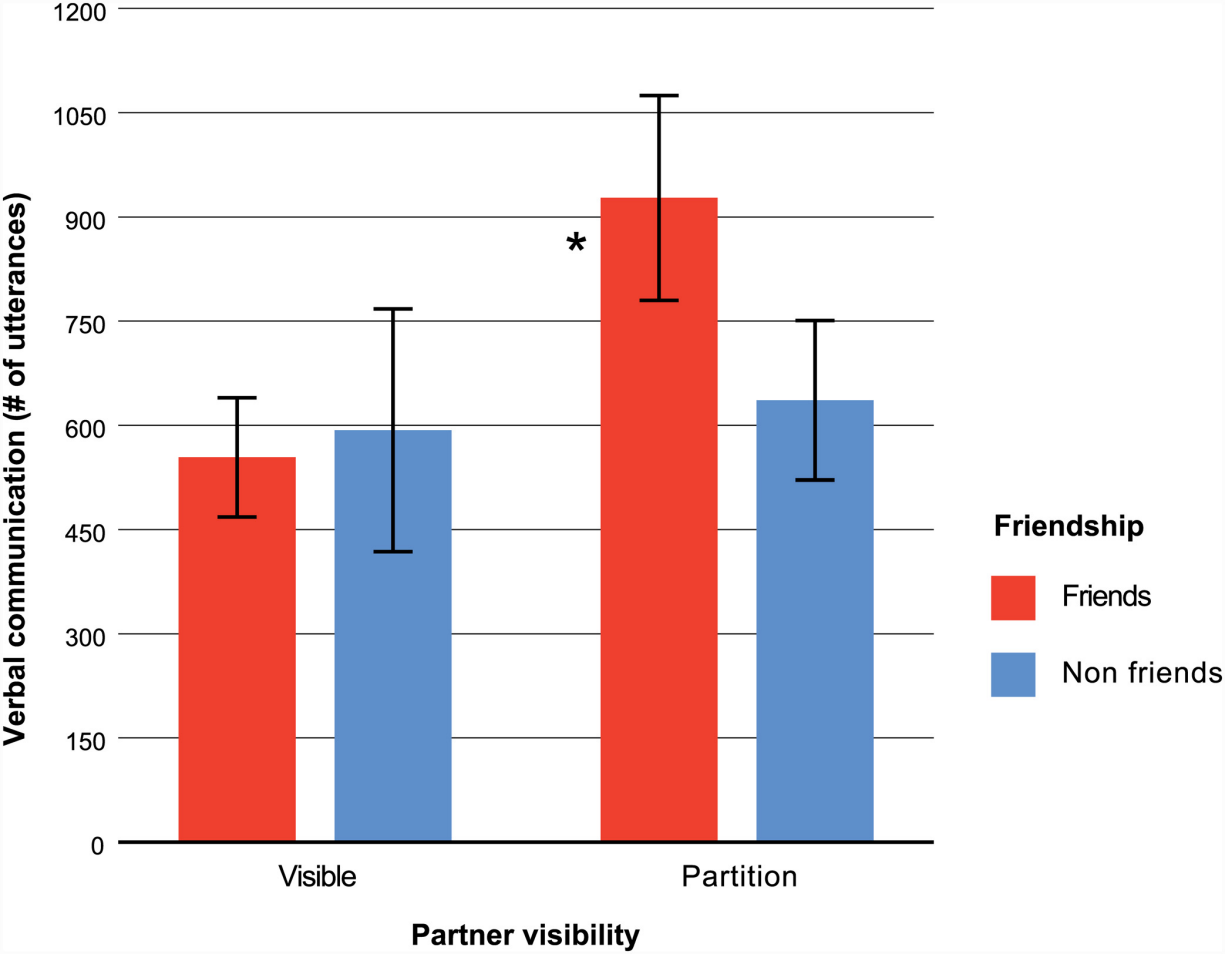
Mean verbal communication as a function of friendship and partner visibility. Verbal communication values indicate the total number of distinct utterances (i.e., words and non-linguistic utterances such as “uh huh”) made by team members during the team task. Teams of friends separated by a partition communicated at the highest rate. Error bars represent 95% confidence intervals around each mean. The asterisk denotes a significant interaction between friendship and partner visibility.

Verbal communication, on the other hand, was negatively associated with collaborative efficiency [r = -.32, p = .01], suggesting that pairs who communicated more while working together during the team task were less efficient. We further investigated this negative association between collaborative efficiency and verbal communication with a 2 X 2 between groups ANOVA on verbal communication with the factors of friendship (friends, non-friends) and partner visibility (visible, partition). Shown in Fig 4, this analysis revealed that teams of friends who were unable to see one another used the most verbal communication. This was demonstrated in main effects of friendship [F(1, 62) = 4.16, p = .046, η_p_^2^ = .06] and partner visibility [F(1, 62) = 11.34, p = .001, η_p_^2^ = .15], and an interaction between these two factors [F(1, 62) = 7.12, p = .010, η_p_^2^ = .10]. To test whether this interaction was influenced by the gender composition of teams, we repeated the analysis of verbal communication, with gender as a covariate (coded as 0, 1, or 2 females). The friendship by partner visibility interaction remained significant [F(1, 59) = 6.44, p = .014, η_p_^2^ = .10]. Here the gender covariate did not influence verbal communication on its own, [F(1, 61) = 1.08, p = .302, η_p_^2^ = .02.

Together these results show that partner visibility supports cognitive collaboration among friends. When friends were visible to each other and able to communicate via body language they were more efficient than non-friends. However, when a partition prevented friends from seeing each other, they communicated verbally instead. Because verbal communication was negatively associated with collaborative efficiency, this switch from nonverbal to verbal communication reduced the efficiency of cognitive collaboration among friends to the same lower level as non-friends.

### The collaborative efficiency advantage does not result from psychophysiological arousal. Arousal does not mediate the effects of friendship and partner visibility on collaborative efficiency

The HR and SCR data were re-sampled offline to 32 Hz. No data filtering was used during the following analyses. Multiple Bonferroni corrected paired sample t-tests evaluated whether psychophysiological arousal levels differed when pairs completed the task as a team versus when each individual completed the task alone. Changes in HR and SCR following an auditory startle were tested to confirm the sensitivity of psychophysiological measures. The results of these analyses are reported below.

Baseline measures of HR and SCR were computed by averaging these values during the 10 sec before the auditory startle was played during both the team and individual tasks. Following the auditory startle to a maximum of 60 sec, the highest HR and SCR were observed on average 2 sec and 15 sec after the auditory startle, respectively. The values at these time points were used to compute the increase in HR and SCR from the auditory startle in each social condition.

HR was higher when two people collaborated [M = 79.19 bpm] than when they worked alone [M = 78.17 bpm; t(61) = -3.44, p < .001, r = .40]. The auditory startle produced an increase in HR when people worked alone [t(61) = -3.42, p < .001, r = .40] and when they worked as a team [t(61) = -3.13, p = .003, r = .37]. There was no difference in SCR when pairs completed the task as a team [M = 6.03 μS] versus completed the task alone [M = 5.30 μS; t(61) = -0.92, p = .361, r = .12]. The auditory startle produced an increase in SCR when people worked alone [t(61) = -10.95 p < .001, r = .81] and when they worked as a team [t(61) = -8.47, p < .001, r = .74].

Concerning the relations between psychophysiological arousal and the factors of friendship and partner visibility, HR did not differ as a result of these factors [all ps > .13], while SCR was lower in friends than non-friends during the team task [F(1, 58) = 6.46, p = .014, partial η^2^ = .10]. No other SCR effects were significant [ps > .20].

To test whether the interaction we observed between partner visibility and friendship was confounded by variation in HR or SCR, we repeated the analysis of collaborative efficiency, but this time entering HR and SCR as covariates. The original interaction [F(1, 68) = 5.50, p = .022, η_p_^2^ = .08] was still significant when HR was included as a covariate [F(1, 55) = 4.04, p = .049, η_p_^2^ = .07], when SCR was included as a covariate [F(1, 55) = 4.31, p = .043, η_p_^2^ = .07], and when both were included simultaneously, [F(1, 54) = 4.18, p = .046, η_p_^2^ = .07]. Moreover, these covariates did not account for a significant proportion of variance in any of the analyses, all *p*-values > .40.

## Discussion

Not all cognitive collaboration results in a joint effort that exceeds the independent contributions of the individual people involved. This study demonstrated that friendship and partner visibility influenced the success of collaboration during a visual enumeration task. By randomly assigning participants to collaborate either with friends or non-friends (i.e., friends of other participants), this study design randomized individual ability while experimentally testing the role of friendship. The same logic applied to collaboration with full visibility of the partner versus collaboration despite a partition that occluded visibility. The results revealed a decisive interaction between these two factors: collaboration by friends was more efficient than collaboration by non-friends, but only when pair members were visible to each other. When partner visibility was prevented with a partition, the efficiency of collaboration between friends and non-friends was reduced to a similar low level.

While this study tested the role of friendship and partner visibility in collaborative success with an experimental design (i.e., participants were assigned randomly to both experimental conditions), uncertainty remains about *which features* of friendship and partner visibility improve collaborative performance. Additional research is needed to understand the mechanisms underlying this effect. We posit that friends who can see one another collaborate efficiently because partner visibility reduces the cognitive load during the visual enumeration task. Communication using body language, which is possible only when pair members can see each other, may be critical to cognitive load reduction. In support of this idea are previous reports that familiarity with an activity reduces the cognitive resources required to complete the activity [38], and familiarity with a face decreases the attentional resources required to process emotional expressions [39]. Familiarity with a collaborator and their style of nonverbal communication therefore has the potential to reduce the cognitive resources required to communicate task relevant information, freeing these resources for the task of enumerating the target objects in the visual display instead.

This study also speaks to the role of verbal communication in successful collaboration. When a partition prevented friends from seeing each other, they compensated by relying on higher levels of verbal communication to share information. Here we reported a negative association between verbal communication and collaborative efficiency, and thus this switch from nonverbal to verbal communication reduced the efficiency of friends to the same low level as non-friends. This finding is akin to previous research reports that teams using either verbal or nonverbal communication were more efficient than teams that used both verbal and nonverbal communication [18].

In addition to showing that friendship and communication influenced the success of cognitive collaboration, the data elucidated the role of social facilitation in collaborative efficiency. HR, but not SCR, was generally higher when two people collaborated than when they worked alone. Yet the data indicated no differences in HR due to the factors of the experiment, and showed lower SCR in teams of friends than non-friends. Importantly, both HR and SCR passed the manipulation check, increasing significantly as a result of the auditory startle across all conditions of the experiment. Considering these findings together, and that speaking itself has been shown to increase HR [31], we conclude that psychophysiological arousal did not explain performance differences between individuals and teams. Teams, but not individuals, conversed during the experiment, and this was sufficient to account for the approximate 1bpm HR increase during team performance.

While the finding of lower SCR in teams of friends than non-friends runs counter to the idea that heightened psychophysiological arousal underlies the efficiency advantage of teams, it may suggest an alternative mechanism through which friendship leads to collaborative success. Friends may benefit from their lower levels of arousal compared to non-friends, as instructions to relax have been reported to improve moderately difficult visual searches [40]. However, the relation between arousal and cognition is complex (for a review see [41]) so this suggestion should be interpreted with caution.

Although this study was not designed to investigate the contributions of gender to collaborative success, the analyses of covariance we conducted indicated several important findings. First, the presence of one or more females on a team contributed positively, as indicated by a significant relation between gender composition (coded as 0, 1, or 2 females) and the magnitude of the collaborative benefit. Second, the data showed a significant interaction between friendship and partner visibility, even after the influence of gender had been removed from consideration. Third, there was no measurable influence of gender on the degree to which friends increased their verbal communication when separated by a partition. The tendency for teams to use more verbal communication when not visible to each other applied equally well to both females and males.

Although there has been much previous research on gender differences in cognition, very little of it has examined gender differences in *collaborative* cognition. One study reported no gender difference in spatial and verbal collaborative memory performance [42]. Another showed that collaborative success was correlated positively with the proportion of females in the groups, although this difference was mediated by the higher social sensitivity or females compared to males (Woolley et al., 2010). The finding in this experiment that the presence of one or more females on a team contributed positively to collaborative success is consistent with the latter finding, although additional research is required to understand the mechanisms by which gender influences collaborative success.

The findings of this study demonstrate that partner visibility interacts with friendship to influence the efficiency of a cognitive collaboration. Importantly, this study’s experimental design elucidates the structure of the relations between these factors. It eliminates the possibility of the reverse direction of influence (i.e., successful task performance influencing the nature of the social interaction) and the possibility that a third factor, such as social intelligence, was responsible for both the quality of the social interaction and team success. While important in their own right, we hope these findings lay the groundwork for further investigation of the mechanisms that underlie effective cognitive collaboration.

## Supporting Information

S1 Dataset (XLSX)
